# Let Us Transform Traits Before Aggregating Them!

**DOI:** 10.1002/ece3.72686

**Published:** 2025-12-18

**Authors:** Zoltán Botta‐Dukát

**Affiliations:** ^1^ HUN‐REN Centre for Ecological Research Vácrátót Hungary

**Keywords:** data transformation, Jensen's inequality, log‐transformation, quadratic approximation, traitsaggregation of traits

## Abstract

Trait values are often log‐transformed during the analyses. When measurements are aggregated at the species level, it matters whether we transform the values before or after aggregation. Since mechanisms creating relationships between traits act at the individual level rather than the species level, transformed values should be aggregated, contrary to the present practice.

## Graphical abstract

Global studies of the relationship between traits typically use trait data aggregated by species. To capture non‐linear relationships, the trait values are often transformed in these studies. Due to Jensen's inequality, transforming species‐level means and averaging transformed individual measurements leads to different results. If the relationship is created by a mechanism operating at the individual level, traits should be transformed before averaging, while the present practice is the opposite (i.e., raw traits are averaged before transformation).

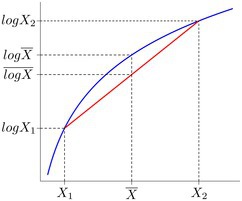

1

The development of trait databases (e.g., Wheeler et al. 2007; Kleyer et al. 2008; Klimešová and De Bello 2009; Kattge et al. 2011, 2020; Sonkoly et al. 2023; Su et al. 2024) facilitated global studies of the relationship between traits (e.g., Diaz et al. 2004; Wright et al. 2004; Díaz et al. 2016; Thomas et al. 2020; Carmona et al. 2021; Weigelt et al. 2021). Although traits, *per definitionem*, are measured at the individual level (Violle et al. 2007), these studies typically use trait data aggregated by species. A central assumption of using species averages is that the aggregated data capture the majority of trait variation (Cordlandwehr et al. 2013). Losing information on intraspecific variation is only one of the issues when aggregated data are used. This paper aims to call attention to a less obvious problem: the relationships may be distorted when data transformation is applied to aggregated data.

During the analysis, trait values are often log‐transformed. There are two possibilities: (1) calculate species‐level means first and then transform them, or (2) transform individual measurements before averaging. If the transformation is non‐linear (changing measurement unit is a linear transformation), the two approaches result in different species‐level transformed values due to Jensen's inequality (Ruel and Ayres 1999).

Let us denote the trait value measured on the *j*th individual by $x_{j}$, the species level mean of untransformed trait values by $\bar{x}$, and the applied transformation by $f\left(\right)$. Applying quadratic approximation (approximation by 2nd‐order Taylor polynomial), the relationship between transformed species‐level traits calculated by the two above‐mentioned ways is the following (see proof in the Appendix A):

(1) $$\bar{f\left(x\right)}\approx f\left(\bar{x}\right)+f^{\prime \prime }\left(\bar{x}\right)\frac{\sum_{j}{\left(x_{j}-\bar{x}\right)}^{2}}{2n}$$

where *n* is the number of measurements on species, and $f^{\prime \prime }\left(x\right)$ is the second derivative of $f\left(x\right)$. Specifically, for log‐transformation:

(2) $$\bar{\ln\left(x\right)}\approx \ln\left(\bar{x}\right)-\frac{1}{{\bar{x}}^{2}}\frac{\sum_{j}{\left(x_{j}-\bar{x}\right)}^{2}}{2n}$$

In this case, the difference is higher when the ratio of within‐species variance and the squared species‐level mean is higher.

Considering a single trait, we cannot decide which way of calculation is better. We can only say that they differ. The situation changes if relationships between traits are analyzed. Let us assume that there is a non‐linear relationship between two traits (*x* and *y*) at the measurement level, which becomes linear after applying appropriate transformations:

(3) $$f\left(y_{j}\right)=a+\mathrm{bg}\left(x_{j}\right)$$

The simplest example is the power function:

(4) $$y_{j}={\mathrm{ax}}_{j}^{b}$$

It becomes linear after the log transformation of both traits:

(5) $$\ln\left(y_{j}\right)=\ln\left(a\right)+b\ln\left(x_{j}\right)$$

The same relationship can be observed between the expected values:

(6) $$E\left[f\left(y\right)\right]=E\left[a+\mathrm{bg}\left(x\right)\right]=a+b\;E\left[g\left(x\right)\right]$$

And also between means if both traits are measured on the same set of individuals:

(7) $$\bar{f\left(y\right)}=\frac{\sum_{j}f\left(y_{j}\right)}{n}=a+b\frac{\sum_{j}g\left(x_{j}\right)}{n}=a+b\bar{g\left(x\right)}$$

Equation (7) remains approximately valid if traits are measured on different sets of individuals because means are approximations of expected values (cf. Equation 6).

However, the relationship is more complicated when the averaged trait values are transformed. Substituting the quadratic approximation (Equation 1) into Equation (7):

(8) $$f\left(\bar{y}\right)+f^{\prime \prime }\left(\bar{y}\right)\frac{\sum_{j}{\left(y_{j}-\bar{y}\right)}^{2}}{2n_{y}}\approx a+b\left[g\left(\bar{x}\right)+g^{\prime \prime }\left(\bar{x}\right)\frac{\sum_{k}{\left(x_{k}-\bar{x}\right)}^{2}}{2n_{x}}\right]$$

Note that different indexing (*j* and *k*) was used for the two traits to emphasize that they were not necessarily measured on the same individuals. For the same reason, sample sizes are denoted by *n*_*x*_ and *n*_*y*_, indicating that they may differ.

After re‐ordering, Equation (8) becomes

(9) $$f\left(\bar{y}\right)\approx a+\mathrm{bg}\left(\bar{x}\right)+{\mathrm{bg}}^{\prime \prime }\left(\bar{x}\right)\frac{\sum_{j}{\left(x_{k}-\bar{x}\right)}^{2}}{2n_{x}}-f^{\prime \prime }\left(\bar{y}\right)\frac{\sum_{j}{\left(y_{j}-\bar{y}\right)}^{2}}{2n_{y}}=a+\mathrm{bg}\left(\bar{x}\right)+C$$

While functions *f*() and *g*(), as well as parameters *a* and *b*, are assumed to be the same for all species, *C* is—*per definitionem*—a species‐specific value. In the lucky case, when *C* is independent of $\bar{x}$, it introduces only a random noise. Probably most often, there is a non‐linear relationship between *C* and $g\left(\bar{x}\right)$ that makes the relationship between $f\left(\bar{y}\right)$ and $g\left(\bar{x}\right)$ also non‐linear. If this non‐linearity is neglected, the parameter estimates will be biased.

In the following, this bias will be illustrated by an example of the relationship between three root traits, where the theoretically expected parameters are known. Assuming cylindrical geometry of fine roots, specific root length (SRL) can be calculated from root diameter (D) and root tissue density (RTD) (Zhang et al. 2024):

(10) $$\mathrm{SRL}=\frac{4}{\pi }{\mathrm{RTD}}^{-1}D^{-2}$$

After log transformation, the relationship becomes linear.

(11) $$\ln\left(\mathrm{SRL}\right)=\ln\left(\frac{4}{\pi }\right)-\ln\left(\mathrm{RTD}\right)-2\ln\left(D\right)$$

Since the relationship emerges from the cylindrical geometry of individual roots, it operates on the level of individual measurements, not on the level of species.

To illustrate this distortion, I downloaded the individual‐level measurements of fine root diameter and fine root tissue density from the TRY database. There were 1920 individuals belonging to 368 species with measurements of both traits. Specific root length of each individual was calculated by Equation (10). Then, raw traits and their log‐transformed values were aggregated to the species level, and equations analogous to Equation (11) were fitted.

When transformed traits were aggregated, the fitted function was:

(12) $$\bar{\ln\left(\mathrm{SRL}\right)}=a+b_{1}\bar{\ln\left(\mathrm{RTD}\right)}+b_{2}\bar{\ln\left(D\right)}$$

This regression results in a perfect fit (*R* ^2^ = 1) and the estimated parameters are equal to the expected ones (*a* = 0.2416; *b*_1_ = −2; *b*_2_ = −1) with standard errors near zero (less than 10^−15^). Note that this perfect fit is due to the way the dataset was created (i.e., SRL values were not directly measured, but calculated from the other two traits). In practice, neither SRL nor RTD is directly measured, but they are calculated from length, diameter, and weight data. Therefore, the perfect fit also appears in real data. However, if the studied traits are measured directly or calculated from non‐overlapping sets of directly measured traits, the regression may not fit perfectly at the individual level. This is due to the independent sampling errors associated with each trait.

When traits were aggregated first and then transformed, the fitted function was

(13) $$\ln\left(\bar{\mathrm{SRL}}\right)=a+b_{1}\ln\left(\bar{\mathrm{RTD}}\right)+b_{2}\ln\left(\bar{D}\right)$$

In this regression, independent variables explained only 63.5% of the variation (*R* ^2^ = 0.635) and the estimated parameters considerably differ from the expectations (*a* = 1.144, se = 0.123; *b*_1_ = −1.552, se = 0.074; *b*_2_ = −0.907, se = 0.044).

Pairwise analysis of trait relationships is more frequent in ecology. Table 1 summarizes the results of such regressions of the three traits applying the two aggregation approaches. Surprisingly, the two approaches differed considerably only in one of the three analyses. The question arises as to what the difference between the two approaches depends on. Let **x**, **y** denote the species‐level trait values aggregating the measurements in one way, and x*, y* aggregating the measurements in the other way. Let us define two other vectors:

(14) $$\begin{array}{c} \theta =x-x^{*}\\ \omega =y-y^{*} \end{array}$$

**TABLE 1:** Summary of the regressions between pairs of three root traits. For explanation of abbreviations see the main text.

Regression	Slope	SE of slope	*R* ^2^
$\bar{\ln\left(\mathrm{SRL}\right)}\sim \bar{\ln\left(\mathrm{RTD}\right)}$	−0.586	0.056	0.228
$\ln\left(\bar{\mathrm{SRL}}\right)\sim \ln\left(\bar{\mathrm{RTD}}\right)$	−0.589	0.061	0.202
$\bar{\ln\left(\mathrm{SRL}\right)}\sim \bar{\ln\left(D\right)}$	−1.378	0.084	0.421
$\ln\left(\bar{\mathrm{SRL}}\right)\sim \ln\left(\bar{D}\right)$	−1.022	0.102	0.214
$\bar{\ln\left(\mathrm{RTD}\right)}\sim \bar{\ln\left(D\right)}$	−0.207	0.028	0.128
$\ln\left(\bar{\mathrm{RTD}}\right)\sim \ln\left(\bar{D}\right)$	−0.205	0.029	0.120

Hereafter, I will refer to these vectors as bias, and to **x**, **y** as unbiased values. The estimated slope of the **x~y** regression is

(15) $$\hat{b}\left(x,y\right)=\frac{\mathrm{cov}\left(x,y\right)}{\mathrm{var}\left(x\right)}$$

while the estimated slope of the x* ~ y* regression is

(16) $$\hat{b}\left(x^{*},y^{*}\right)=\frac{\mathrm{cov}\left(x^{*},y^{*}\right)}{\mathrm{var}\left(x^{*}\right)}=\frac{\mathrm{cov}\left(x+\theta ,y+\omega \right)}{\mathrm{var}\left(x+\theta \right)}=\frac{\mathrm{cov}\left(x,y\right)+\mathrm{cov}\left(x,\omega \right)+\mathrm{cov}\left(\theta ,y\right)+\mathrm{cov}\left(\theta ,\omega \right)}{\mathrm{var}\left(x\right)+\mathrm{var}\left(\theta \right)+2\mathrm{cov}\left(x,\theta \right)}$$

It can be seen that the difference between the two slopes depends on the variance–covariance matrix of the unbiased values (**x**, **y**) and species‐level biases $\left(\theta ,\omega \right)$. The difference does not directly depend on the mean bias. However, practically, the higher mean often results in higher between‐species variation of bias, leading to a higher difference between slope estimates.

We can reach the same conclusion for the correlation coefficient:

(17) $$\begin{array}{c} r\left(x,y\right)=\frac{\mathrm{cov}\left(x,y\right)}{\sqrt{\mathrm{var}\left(x\right)\mathrm{var}\left(y\right)}}\\ r\left(x^{*},y^{*}\right)=\frac{\mathrm{cov}\left(x^{*},y^{*}\right)}{\sqrt{\mathrm{var}\left(x^{*}\right)\mathrm{var}\left(y^{*}\right)}}=\frac{\mathrm{cov}\left(x+\theta ,y+\omega \right)}{\sqrt{\mathrm{var}\left(x+\theta \right)\mathrm{var}\left(y+\omega \right)}}=\frac{\mathrm{cov}\left(x,y\right)+\mathrm{cov}\left(x,\omega \right)+\mathrm{cov}\left(\theta ,y\right)+\mathrm{cov}\left(\theta ,\omega \right)}{\sqrt{\left(\mathrm{var}\left(x\right)+\mathrm{var}\left(\theta \right)+\mathrm{cov}\left(x,\theta \right)\right)\left(\mathrm{var}\left(y\right)+\mathrm{var}\left(\omega \right)+\mathrm{cov}\left(y,\omega \right)\right)}} \end{array}$$

It means that there is no simple general rule where the difference between the two approaches is considerable. If individual values are not known, but an estimate of the intraspecific variance for each species is available, then an approximate correction can be made using Equation (2). This is also suitable for estimating the magnitude of the difference between the two aggregation approaches.

The theoretical considerations proved, and the example illustrated that inappropriate order of transformation and aggregation of trait values may lead to (1) less perfect fit and, consequently, underestimation of the strength of the relationship, (2) biased parameter estimates, and (3) higher uncertainty of parameter estimates that leads to increased variation of parameter estimates among studies. In the example, it was illustrated by log‐transformation, but this is true for any non‐linear transformation (e.g., square root, arcsine) because, due to Jensen's inequality, the transformed average value differs from the average of the transformed values.

The typical order is aggregating trait values first and then transforming the aggregated values. It is the appropriate order only if the processes creating the relationships between traits are acting at the species level. Since these processes are more probably acting at the level of individuals, I suggest that the opposite order (i.e., averaging the transformed trait values) is more appropriate in most cases. One could argue that the optimal order of aggregation and transformation depends on the purpose of the study. In studies focusing on between‐species differences (e.g., coordinated evolution of traits), the units are species; therefore, transformation could be applied at the species level (i.e., after aggregation). I do not share this opinion. In my view, it is not the purpose of the study, but the organizational level where the mechanisms that create correlations between traits operate that determines the optimal order. Geometric constraints creating the relationship among the three root traits in the example clearly act at the individual measurement level (i.e., at the level of the measured root section). Correlation between traits may also appear when they could change independently, but some combination of their values is disadvantageous. In this case, individuals with these disadvantageous combinations of trait values are selected out due to their higher mortality and/or lower fecundity. For example, four hydraulic traits, minimum leaf water potential (*ψ*_min_), P50 (water potential that causes 50% reduction in stem hydraulic conductivity), maximum stem specific conductivity (K_S_), and Huber value (the ratio of cross‐sectional sapwood area relative to leaf area) cannot be changed independently without endangering the safe operation in dry conditions, and without allowing effective photosynthesis in good water supply. For example, four hydraulic properties, minimum leaf water potential (*ψ*_min_), P50 (the water potential that causes a 50% reduction in stem hydraulic conductivity), maximum stem specific conductivity (KS), and Huber value (the ratio of sapwood cross‐sectional area to leaf area) cannot be changed independently without hindering efficient photosynthesis with a good water supply or compromising safe operation in dry conditions (Sanchez‐Martinez et al. 2020). Although Sanchez‐Martinez et al. (2020) studied the correlations among these hydraulic traits at the species level, the selection against unfavorable combinations of trait values operates at the individual level through low fecundity or high mortality. Therefore, in my opinion, the transformed values should have been aggregated in this study.

In large‐scale studies, trait data are typically obtained from databases. Some databases—for example, FloraVeg.EU (Chytrý et al. 2024), PLADIAS (Chytrý et al. 2021), or PADAPT (Sonkoly et al. 2023)—provide only aggregated data. However, TRY, the largest plant trait database, stores data at the aggregation level at which it was published. Thus, it contains a lot of individual measurements. I recommend using these measurements instead of aggregated values whenever possible, and encourage data providers to publish the individual measurements, even if the analyses they use aggregated data.

## Author Contributions

**Zoltán Botta‐Dukát:** conceptualization (lead), formal analysis (lead), writing – original draft (equal), writing – review and editing (equal).

## Funding

This research was supported by NKFIH (project number K138674).

## Conflicts of Interest

The author declares no conflicts of interest.

## Data Availability

Data and code are available at https://doi.org/10.5281/zenodo.17961799.

## Appendix A Approximation of the Mean and Expected Value of the Function by 2nd‐Order Taylor Polynomials

The th‐order Taylor polynomial is an approximation of a ‐times differentiable function around a given point by a polynomial of degree

(A1)
$$f\left(x\right)\approx f\left(a\right)+\sum_{i=1}^{k}\frac{f^{\left(i\right)}\left(a\right)}{i!}{\left(x-a\right)}^{i}$$

where $f^{\left(i\right)}\left(a\right)$ denotes the *i*th derivative of *f* evaluated at point *a*. Hereafter, we use the 2nd‐order Taylor polynomial or quadratic approximation.

We want to approximate the mean of *n* transformed trait values:

(A2)
$$\bar{f\left(x\right)}=\frac{\sum f\left(x_{i}\right)}{n}$$

Applying polynomial approximation around the mean of untransformed values:

(A3)
$$\bar{f\left(x\right)}\approx f\left(\bar{x}\right)+f^{\prime }\left(\bar{x}\right)\frac{\sum \left(x_{i}-\bar{x}\right)}{n}+f^{\prime \prime }\left(\bar{x}\right)\frac{\sum {\left(x_{i}-\bar{x}\right)}^{2}}{2n}$$

Since the sum of the departures from the mean is zero, this equation reduces to Equation (1) in the main text.

In the following, an alternative derivation of Equation (8) is presented. When *x* is a random variate with a known expected value $E\left[x\right]$ and population variance $D^{2}\left[x\right]=E\left[{\left(x-E\left[x\right]\right)}^{2}\right]$, the quadratic approximation of the expected value of *f*(*x*) around $E\left[x\right]$ is analogous to Equation (1):

(A4)
$$E\left[f\left(x\right)\right]\approx f\left(E\left[x\right]\right)+f^{\prime }\left(E\left[x\right]\right)E\left[x_{i}-E\left[x\right]\right]+f^{\prime \prime }\left(E\left[x\right]\right)\frac{E\left[{\left(x_{i}-E\left[x\right]\right)}^{2}\right]}{2}=f\left(E\left[x\right]\right)+f^{\prime \prime }\left(E\left[x\right]\right)\frac{D^{2}\left[x\right]}{2}$$

Substituting the quadratic approximation (Equation 17) into Equation (6) and reordering results in:

(A5)
$$f\left(E\left[y\right]\right)\approx a+\mathrm{bg}\left(E\left[x\right]\right)+{\mathrm{bg}}^{\prime \prime }\left(E\left[x\right]\right)\frac{D^{2}\left[x\right]}{2}-f^{\prime \prime }\left(E\left[y\right]\right)\frac{D^{2}\left[y\right]}{2}=a+\mathrm{bg}\left(E\left[x\right]\right)+C$$

If a priori known expected values and variances in Equation (16) are replaced by their sample estimates, Equation (8) is the result.
